# Semen parameters in men recovered from COVID-19: a systematic review and meta-analysis

**DOI:** 10.1186/s43043-021-00089-w

**Published:** 2021-12-02

**Authors:** Sagar Tiwari, Niranjan KC, Sajan Thapa, Anuja Ghimire, Sanjeev Bijukchhe, Guru Sharan Sah, Ronny Isnuwardana

**Affiliations:** 1 Department of Medicine, Bharatpur Central Hospital, Chitwan, Nepal; 2 Emergency Unit, Sumeru City Hospital, Kathmandu, Nepal; 3 Patan Academy of Health Sciences, Lalitpur, Nepal; 4 Department of Medical Oncology Unit II, B P Koirala Memorial Cancer Hospital, Chitwan, Nepal; 5 Department of Public Health and Community Medicine, Mulawarman University, Samarinda, Indonesia

**Keywords:** COVID-19, Infertility, Male, Meta-analysis, Semen, SARS-CoV-2

## Abstract

**Background:**

The novel beta-coronavirus disease (COVID-19) has infected millions of people globally with high risk among males than females. However, the effect of COVID-19 andrology is still a subject of dispute. We planned to analyze the overall consequences of COVID-19 on semen parameters and male sex hormones.

**Main text:**

Systematic search was performed on MEDLINE and Scopus database until 11 June 2021. We included observational studies, which reported mean ± standard deviation of the semen parameters and serum sex hormones of those reproductive-aged males recovered from COVID-19 and controls who did not suffered from COVID-19. We used Random-effect model to pool the studies, as heterogeneity was present. The *Q* test and *I*^2^ evaluated heterogeneity. All articles were assessed with their quality and publication bias.

We assessed 966 articles for eligibility and found seven eligible studies. These 7 studies included 934 participants with mean age 37.34 ± 10.5 years. Random-effect model meta-analysis showed that men who recovered from COVID-19 had semen parameters less than those who had not suffered from COVID-19. The overall mean difference (MD) [95% confidence interval (CI)] in semen volume, sperm concentration, sperm number, and progressive sperm motility was − 0.20 (− 0.45, 0.05) ml, − 16.59 (− 34.82, 1.65) millions/ml, − 45.44 (− 84.56, − 6.31) millions per ejaculate, − 1.73 (− 8.20, 4.75) percentage respectively. Considering sex hormones, luteinizing hormone and prolactin levels were higher among those recovered with a significant MD (95% CI) of 3.47 (1.59, 5.35)U l^−1^ and 3.21 (1.71, 4.72)ng ml^−1^ respectively.

**Conclusion:**

We found that COVID-19 affects both semen parameters and sexual hormones. However, the mechanism for testicular involvement remains doubtful.

**Trial registration:**

PROSPERO CRD42021259445

**Supplementary Information:**

The online version contains supplementary material available at 10.1186/s43043-021-00089-w.

## Background

The novel beta-coronavirus, also called “severe acute respiratory syndrome coronavirus 2” (SARS-CoV2) has infected millions globally, since first reported from Wuhan, China, on 31 December 2019 [1]. Its common symptoms include dry cough, fever, malaise, dyspnea, and fatigue. Due to its rapid spread and multiple organ damage, concerned authorities and healthcare workers have taken significant measures to address this pandemic [2, 3].

Studies have shown the disease to affect various systems of the body. Recently, SARS-CoV-2 RNA has been found in the semen of a COVID-19 patient - however, this has been a disputed issue. Li et al. [4] conducted a study on male patients with COVID-19 and those recovering from the disease, identified SARS-CoV-2 in semen. In contrast, other studies on acutely infected and recovered patients of COVID-19 did not report SARS-CoV-2 RNAs in the semen [5, 6]. Studies have shown the utilization of angiotensin-converting enzyme II (ACE2) as a receptor by SARS-CoV2 to enter the host cell [7]. An expression profile of a single-cell-human RNA found that Leydig, Sertoli, and spermatogonial cells are enriched with ACE2 [8].

A study from Yang et al. revealed COVID-19 patients with an impairment in testicular histology, morphological changes in Sertoli cells, decreased Leydig cells, and lymphocytic inflammation [9]. These changes reflect the unfavorable effect of COVID-19 on the male reproductive system. A longitudinal study among those recovered from COVID-19 observed a negative impact on sperm quality, although these were likely to be reversible [10]. However, Holtman et al. found that adverse effects on sperm count and sperm motility were only detected in hospitalized patients but not in subjects who have recovered after mild symptoms [11]. Overall, there are varying results regarding the sexual hormone secretion among those recovered from SARS-CoV2 [10, 12, 13].

Recent evidence is inconclusive regarding the effect of COVID-19 on the male reproductive system. Exploring the overall impact of COVID-19 on the semen parameter and male reproductive hormones may also contribute to further investigation into male reproductive endocrinology. To our knowledge, a meta-analysis on semen analysis among patients recovered from COVID-19 lacks to date. Therefore, we aim to investigate the overall difference in semen parameters and male sex hormones between those recovered from COVID-19 and those not infected.

## Methodology

We registered this study in the PROSPERO (CRD42021259445) before conducting the research. We followed the PRISMA guidelines to perform and report this systematic review and meta-analysis [14]. We performed a comprehensive literature search of PubMed/MEDLINE, Scopus databases by two independent reviewers (ST and AG) from inception until 11 June 2021 to find the relevant papers based on the PICO criteria mentioned as follows. The population include reproductive-aged male. Participants who recovered from COVID-19 were in intervention group, while those who did not suffer from COVID-19 were under control group. The outcome assessed was the analysis of semen and sex hormones. For semen analysis, we evaluated and collected semen volume milliliter (mL), total sperm number (millions per ejaculate), sperm concentration (millions/mL), progressive motility (%), sperm vitality (%), and sperm morphology (%). And for sex hormones level, follicle-stimulating hormones (FSH), luteinizing hormone (LH), progesterone, testosterone, prolactin, and inhibin were analyzed where possible. Semen samples were obtained by masturbation and then ejaculated into sterile containers [10, 11, 15]. Sperm was analyzed in accordance with WHO laboratory manuals [16]. For hormonal analysis, the peripheral blood sample was used [12, 13, 17]. We also explored the references in the retrieved articles. The search terms were “COVID 19”, “corona virus”, coronavirus, “severe acute respiratory syndrome coronavirus 2”, SARS CoV2”, semen, sperm, and seminal. The two reviewers screened all the studies by title, abstract and keywords. When in need of more information, full-text was referred. Two independent reviewers extracted data from the study selected and stored it in the spreadsheet electronically. The information included the first author’s name, date of publication, design, location, sample size, characteristics of study participants, including mean age, mean BMI (body mass index), duration of the symptom. Total participants (N), mean and standard deviation (SD) of all these mentioned parameters were extracted for those recovered from COVID-19 and healthy/non-COVID groups. Studies were excluded if published in a language that was untranslatable, multiple publications, or those with incomplete data after 2 attempts to contact the authors.

Two independent reviewers (ST and RS) assessed the risk of bias for each study using the Newcastle Ottawa scale (NOS) adapted for cross-sectional, case-control, and cohort studies [18]. This scale rated the quality of the study in three main domains for each study design: selection, comparability, and outcome. The maximum score was nine. Disagreements were solved by a discussion between two reviewers (SB, AG), which was later reviewed by the principal investigator (GSS).

For pooling effect sizes, direct meta-analysis was performed, including unstandardized mean difference (MD) of semen parameters and sex hormones in subjects recovered from COVID-19 and non-COVID recovered patients. *Q* test and *I*^2^ statistics were applied to assess heterogeneity between studies [19]. The presence of heterogeneity was claimed if a *p* value of the *Q* test was > 0.1 or the *I*^2^ statistic was less than 25%. Mean differences (MD) were pooled across studies using the inverse variance method if they were homogeneous; otherwise, DerSimonian and Laird method was used. Publication bias was assessed by a funnel plot and Egger’s test [20]. A *p* value of < 0.05 was considered statistically significant except for heterogeneity it was < 0.1. We performed all the analyses using STATA software, version 16.1 by StataCorp. (College Station, TX).

## Results

**Fig. 1:**
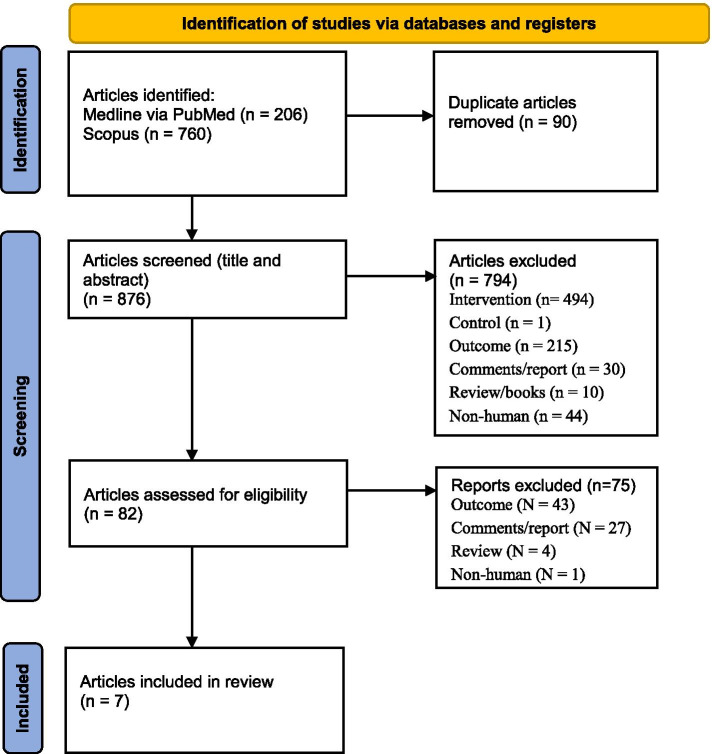
PRISMA diagram

We identified 206 and 760 studies from MEDLINE via PubMed and Scopus, respectively (Fig. 1). Of them, 7 studies met our eligibility criteria. Characteristics of the 7 studies are described in Table 1. There were 5 [12, 13, 17, 21, 22], 1 [10], and 1 [11] cross-sectional, case-control, and cohort studies, respectively. The participants either recovered from COVID-19 or were without a history of COVID-19 infection and had a mean age and BMI of 37.34 ± 10.5 and 25.6 ± 0.65, respectively. These studies were conducted in China [10, 13, 17, 22], Turkey [12, 21], and Germany [11]. The risks of bias assessment of the studies are presented in the Supplemental Table 1. All the included studies were of good quality.

**Table 1:** Basic study characteristics

Author (year)	Country	Design	Mean age	Mean BMI	Outcomes	
					Semen analysis	Sex hormones
Erbay (2021) [21]	Turkey	Cross-sectional	30.68	25.28	Semen volume, sperm concentration, total sperm number, progressive sperm motility, vitality	
Guo (2021) [10]	China	Case-control	27.55		Semen volume, sperm concentration, total sperm number, progressive sperm motility, sperm motility, vitality	Estradiol, FSH, LH, testosterone, prolactin, inhibin
Holtman (2021) [11]	Germany	Cohort	38.2	25.2	Semen volume, sperm concentration, total sperm number, progressive sperm motility, sperm motility	Estradiol
Ma (2021) [13]	China	Cross-sectional	38.2			FSH, LH, testosterone
Raun (2021) [22]	China	Cross-sectional	30.8		Semen volume, sperm concentration, total sperm number, progressive sperm motility, sperm motility	
Temiz (2021) [12]	Turkey	Cross-sectional	36.6	26.6	Total sperm number, progressive sperm motility, sperm motility	FSH, LH, testosterone, prolactin
Xu (2021) [17]	China	Cross-sectional	59.2	25.5		Estradiol, FSH, LH, testosterone

Units of semen analysis according to WHO fifth edition, semen volume (ml), sperm concentration (× 10^6^ ml^−1^), total sperm count (millions per ejaculate), progressive sperm motility (%), sperm motility (%), sperm vitality (%), WBC (× 10^9^/L). Unit of sex hormones, estradiol (pg ml^−1^), FSH (U l^−1^), LH (U l^−1^), testosterone (ng ml^−1^), prolactin (ng ml^−1^)

*FSH* follicle stimulating hormone, *LH* luteinizing hormone

Semen analysis was performed in the following components:

### Semen volume

**Fig. 2:**
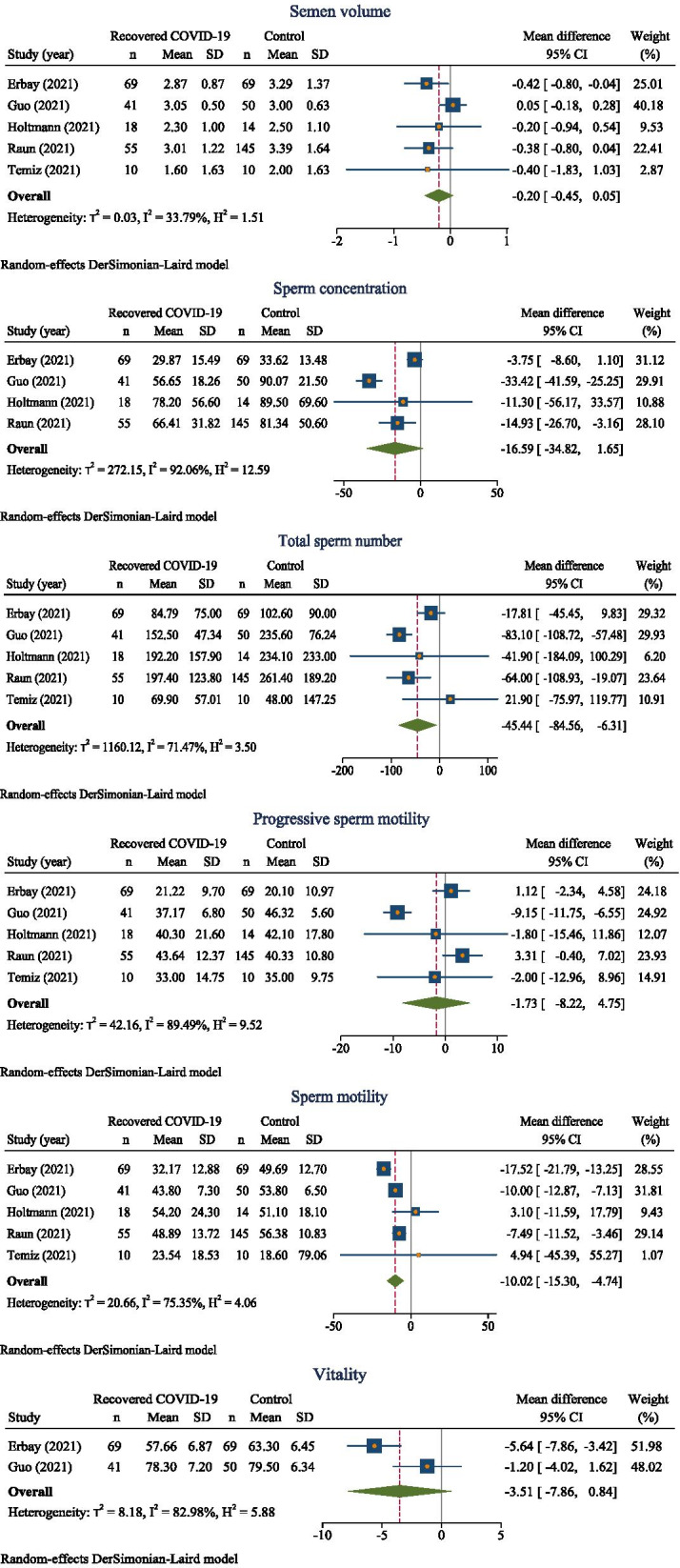
Forest plots of estimation of pooled MDs of semen analysis between those recovered from COVID-19 and those without COVID-19

According to the meta-analysis from 5 studies [10–12, 21, 22], subjects recovered from COVID-19 have semen volume about 0.2 ml less than those without COVID-19 (overall MD − 0.20; 95% CI − 0.45, 0.05) (Fig. 2). However, MDs between-study were moderately varied (*p* = 0.20, *I*^2^ = 33.79%). The funnel plot was symmetrical, corresponding to Egger’s test (coefficient = − 1.3, standard error (SE) = 0.93, *p* = 0.17) (Supplemental Figure 1).

### Sperm concentration

Subjects who recovered from COVID-19 had sperm concentrations about 16.59 × 10^6^ ml less than those without COVID-19 (overall MD − 16.59, 95% CI − 34.82, 1.65) (Fig. 2). This MDs between 4 studies [10, 11, 21, 22] were highly varied (*p* < 0.01, *I*^2^ = 92.06%). The funnel plot was asymmetrical, corresponding to the Egger’s test (coefficient = − 2.19, SE = 0.959, *p* = 0.022). A contour-enhanced funnel plot showed the missing study in the non-significant area indicating asymmetry might be due to publication bias. However, meta-trim suggested no missing studies concluding that the asymmetry was not due to missing study, but rather heterogeneity (Supplemental Figure 2).

### Total sperm number

MD from 5 studies [10–12, 21, 22] were highly varied (*p* = 0.02, *I*^2^ = 71.47%). There was statistically significant pooled MD (95% CI) of − 45.44 (− 84.56, − 6.31) suggesting that those recovered form COVID-19 have sperm number less than 45.4 × 10^6^ per ejaculation (Fig. 2). The funnel plot was symmetrical corresponding the Egger test (coefficient = 0.95, SE = 0.91, *p* = 0.29) (Supplemental Figure 3).

### Progressive sperm motility

According to the meta-analysis from 5 studies [10–12, 21, 22], progressive sperm motility of those subjects recovered from COVID-19 was 1.73% less than those without COVID-19 (overall MD − 1.73; 95% CI − 8.20, 4.75; *p* = 0.20, *I*^2^ = 89.49%) (Fig. 2). The funnel plot was symmetrical, corresponding to Egger’s test (coefficient = 1.77, SE = 0.93, *p* = 0.06) (Supplemental Figure 4).

### Sperm motility

MD from 5 studies [10–12, 21, 22] were highly varied (*p* ≤ 0.001, *I*^2^ = 75.35%). There was statistically significant pooled MD (95% CI) of − 10.02 (− 15.30, − 4.74) (Fig. 2) suggesting that those recovered form COVID-19 have total sperm motility less than about 10%. The funnel plot was symmetrical corresponding the Egger test (coefficient = − 1, SE = 0.81, *p* = 0.21) (Supplemental Figure 5).

### Vitality

MD from 2 studies [10, 21] were highly varied (*p* = 0.23, *I*^2^ = 82.98%). The pooled MD was − 3.51(− 7.86, 0.84) (Fig. 2).

Considering the male sex hormones,

### FSH

**Fig. 3:**
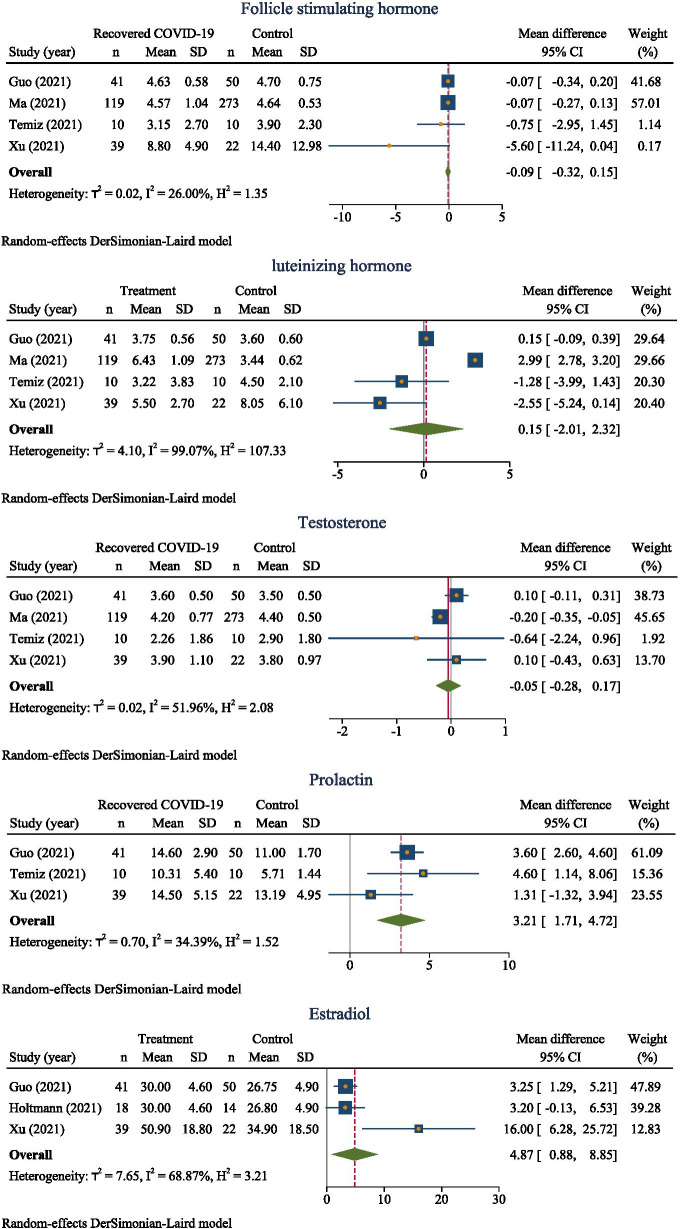
Forest plot of estimation of MDs of sex hormones between those recovered from COVID-19 and those without COVID-19

According to the meta-analysis from 4 studies [10, 12, 13, 17], FSH among those subjects recovered from COVID-19 was 0.09 mIU/mL less than those without COVID-19 (overall MD − 0.09; 95% CI − 0.32, 0.15; *p* = 0.26 *I*^2^ = 26%) (Fig. 3). The funnel plot was symmetrical, corresponding to Egger’s test (coefficient = − 1.34, SE = 0.75, *p* = 0.07) (Supplemental Figure 6).

### LH

Meta-analysis from 4 studies [10, 12, 13, 17] revealed that subject recovered from COVID-19 was 3.47 mIU/mL more than those without COVID-19 (overall MD 0.15; 95% CI − 2.013, 2.316). However, MD between studies were highly varied (*p* = < 0.001, *I*^2^ = 99.07%) (Fig. 3). The funnel plot was asymmetrical corresponding to the Egger’s test (coefficient = − 2.78, SE = 1.74, *P* = 0.11). A contour-enhanced funnel plot showed the missing study in the non-significant area indicating that the asymmetry might be due to publication bias. Therefore, the meta-trim fills method was used which suggests no missing studies (Supplemental Figure 7). Thus, we conclude that the asymmetry was not due to missing study but heterogeneity might be the cause for asymmetry.

### Testosterone

Testosterone among those recovered from COVID-19 is less than that in non-COVID overall MD − 0.051 95% CI (− 0.277, 0.175) (Fig. 3). MD between these 4 studies [10, 12, 13, 17] were moderately varied (*p* = 0.10, *I*^2^ = 51.96%). The funnel plot was symmetrical corresponding to the Eggers test (coefficient = − 0.28, SE = 1.03, *p* = 0.73) (Supplemental Figure 8).

### Prolactin

Prolactin level among those recovered from COVID-19 was more than that among non-COVID-19 groups with overall MD (95% CI) of 3.21 (1.71, 4.72) with presence of low heterogeneity (*p* = 0.21, *I*^2^ = 34.39%) from 3 studies [10, 12, 17] (Fig. 3). This signifies the level of Prolactin was about 3 ng/ml times more among those recovered from COVID-19.

### Estradiol

Estradiol level among those recovered from COVID-19 was more than those non-COVID-19 groups and this MD between 3 studies were moderately varied: overall MD (95% CI) of 4.87 (0.88, 8.85; *I*^2^ = 68.87%, *p* = 0.04) (Fig. 3).

## Discussion

Looking into the effect of COVID-19 on semen and fertility of males recovered from COVID-19, we compared the overall semen analysis and sex hormone profile between the recovered COVID-19 patients and those without COVID-19 infection. Lesser semen volume, sperm concentration, and progressive sperm motility were found among those recovered from COVID-19, although not significant. Similarly, statistically significant MD of sperm number and total sperm mortality between the COVID-19 group and those without COVID-19 infection was observed. Regarding the hormones, statistically significant higher LH and prolactin were found among those COVID-19 patients. However, the overall FSH and testosterone were more among those non-COVID-19 patients although non-significant.

The male reproductive function might be affected by a wide range of viruses including influenza, mumps virus, Zika virus, human immunodeficiency virus (HIV), and even may infect testes [23]. Other than the direct effects of the virus on the testes, there are several other indirect factors such as inflammation, fever, and the dysregulation of the hypothalamic–pituitary–gonadal axis, which may cause the impairment of testosterone secretion or sperm production.

The long-term effect on male fertility by SARS-CoV-2 is still a subject of debate. The study by Guo et al. illustrated that the sperm concentration, total sperm count, and progressive motility following the COVID-19 recovery were increased significantly at the sampling conducted at the median of 84 days than that with the first sampling at 56 days of recovery [10]. As the human spermatogenic cycle is estimated taking 74 days, it can be suggested that the effects of COVID-19 might last for 74 days [24].

The SARS-CoV and SARS-CoV-2 showed almost similarities in the complete genomic sequences,: they both have ACE2 receptor and spike protein (protein S) to enter the host cell. The protein S is filled by a transmembrane serine protease (TMPRSS2). Sertoli cells, Leydig cells, prostatic epithelial cells, and spermatogonia all express TMPRSS2 and ACE-2 receptors [8]. Overall, the way of transmission and genetic similarity of the SARS-COV-2 virus affects males than females (male: female ratio 2.7:1) than SARS-CoV. Hence its effect on male reproductive function is a subject of research [25]. Although the most common manifestation of COVID-19 is pneumonia due to the occupancy of ACE2 containing cells, ACE2 receptors are also present in most organs. As the study revealed that the Sertoli and Leydig cells have a higher level of ACE2 receptor than type II alveolar cells, this puts the testicles at risk to SARS-CoV-2. The virus reaches the lungs by the inhalation of droplets but there are no any direct way to reach the testes and other targeted organs which can be the logic for the respiratory symptoms being more characteristics.

As SARS-CoV-2 was detected in the semen in recent studies, it could be sexually transmitted [10, 11]. Although Machado et al., detected a viral RNA in one case out of 15 study participants, the methodology used was not mentioned. They mentioned that masturbation, as one of the collection methods for semen samples, could cause contamination through hand and even cough. The observed decrease in the semen parameters could be due to the injury from the SARS-COV-2 infection triggered by the immune response in opposition to the seminiferous epithelium [25]. Like most infectious diseases, a decrease in testosterone level can be due to the inflammatory process enhanced by fever, which damages the germ cells by infiltration. It could be even due to a breach on the barrier of blood-testis [26]. The increased level of LH could be due to the compensated hypogonadism [27]. Some of the studies reported that some cytokines as the putative markers of infections [28]. Interleukins-8 is involved in the inflammation of seminal vesicles, prostate ad epididymis, which makes it a marker for male accessory gland infection [29, 30]. Furthermore, the different procedures involved during treatment like; ventilation support, use of sedative drugs, steroids, antibiotics, organ support therapy, and so on and even distress, can affect the function of testis [31]. However, most of our study participants were recovered from COVID-19 infection and were confirmed PCR-negative, the impact of inflammatory process by fever on the semen quality seems to be minor. Also, we should keep in mind that a variety of psychological and environmental factors play a role. The stressful events from COVID-19 ultimately make sex less desirable. Hence, those recovering should also focus on their psychological state and consider the counseling.

However, there are some limitations of this meta-analysis. Most of the included studies were cross-sectional design without any casual verification of COVID-19 infection with the affection of semen parameters and sex hormones, so causal association between them could not be worked out. Heterogeneity was high between included studies. Other clinical characteristics might have also caused heterogeneity but the data were not available for subgroup analysis.

In conclusion, the semen parameters were found to be affected by the SARA-CoV-2. This raises the possibility that SARS-CoV-2 could affect the sexual development and fertility of males. However, more future prospective cohort studies will need to prove and simplify the association.

## Supplementary Information

**Additional file 1: Supplementary Table 1.** Risk of bias assessment of included studies.

**Additional file 2: Supplementary Fig. 1.** Funnel plot for pooling of semen volume. **Supplementary Fig. 2.** Funnel plot for pooling of sperm concentration. **Supplementary Fig. 3.** Funnel plot for total sperm number. **Supplementary Fig. 4.** Funnel plot for progressive sperm motility. **Supplementary Fig. 5.** Funnel plot for sperm motility. **Supplementary Fig. 6.** Funnel plot for Follicle stimulating hormone. **Supplementary Fig. 7.** Funnel plot for Luteinizing hormone. **Supplementary Fig. 8.** Funnel plot for Testosterone.

## Data Availability

Not applicable

## Abbreviations

ACE2: Angiotensin-converting enzyme II
BMI: Body mass index
COVID-19: Corona virus disease
FSH: Follicle-stimulating hormones
HIV: Human immunodeficiency virus
LH: Luteinizing hormone
MD: Mean difference
NOS: Newcastle-Ottawa scale
Protein S: Spike protein
SARS-CoV2: Severe acute respiratory syndrome coronavirus 2
SD: Standard deviation
SE: Standard error
TMPRSS2: Transmembrane serine protease

## References

[1] The Johns Hopkins Coronavirus Resource Center. COVID-19 Map 2021 [updated 15 Aug 2021]. Available from: https://coronavirus.jhu.edu/map.html.

[2] Adams JG, Walls RM (2020). Supporting the health care workforce during the COVID-19 global epidemic. JAMA.

[3] Wong JE, Leo YS, Tan CC (2020). COVID-19 in Singapore—current experience: critical global issues that require attention and action. JAMA.

[4] Li D, Jin M, Bao P (2020). Clinical characteristics and results of semen tests among men with coronavirus disease 2019. JAMA Netw Open.

[5] Pan F, Xiao X, Guo J (2020). No evidence of SARS-CoV-2 in 393 semen of males recovering from COVID-19. Fertil Steril.

[6] Paoli D, Pallotti F, Colangelo S (2020). Study of SARS-CoV-2 in semen and urine samples of a volunteer with positive naso-pharyngeal swab. J Endocrinol Investig.

[7] Zhou P, Yang X-L, Wang X-G (2020). A pneumonia outbreak associated with a new coronavirus of probable bat origin. Nature.

[8] Wang Z, Xu X (2020). scRNA-seq profiling of human testes reveals the presence of the ACE2 receptor, a target for SARS-CoV-2 infection in spermatogonia, Leydig and Sertoli cells. Cells.

[9] Yang M, Chen S, Huang B (2020). Pathological findings in the testes of COVID-19 patients: clinical implications. Eur Urol Focus.

[10] Guo TH, Sang MY, Bai S et al (2021) Semen parameters in men recovered from COVID-19. Asian J Androl. 10.4103/aja.aja_31_21 [published Online First: 13 May 2021] 10.4103/aja.aja_31_21 PMC8451500 33975987

[11] Holtmann N, Edimiris P, Andree M (2020). Assessment of SARS-CoV-2 in human semen-a cohort study. Fertil Steril.

[12] Temiz MZ, Dincer MM, Hacibey I (2021). Investigation of SARS-CoV-2 in semen samples and the effects of COVID-19 on male sexual health by using semen analysis and serum male hormone profile: a cross-sectional, pilot study. Andrologia.

[13] Ma L, Xie W, Li D (2021). Evaluation of sex-related hormones and semen characteristics in reproductive-aged male COVID-19 patients. J Med Virol.

[14] Liberati A, Altman DG, Tetzlaff J (2009). The PRISMA statement for reporting systematic reviews and meta-analyses of studies that evaluate health care interventions: explanation and elaboration. J Clin Epidemiol.

[15] Gacci M, Coppi M, Baldi E (2021). Semen impairment and occurrence of SARS-CoV-2 virus in semen after recovery from COVID-19. Hum Reprod.

[16] World Health Organization (2010). WHO laboratory manual for the examination and processing of human semen.

[17] Xu H, Wang Z, Feng C (2021). Effects of SARS-CoV-2 infection on male sex-related hormones in recovering patients. Andrology.

[18] Zeng X, Zhang Y, Kwong JS (2015). The methodological quality assessment tools for preclinical and clinical studies, systematic review and meta-analysis, and clinical practice guideline: a systematic review. J Evid Based Med.

[19] Thompson SG (2001). Why and how sources of heterogeneity should be investigated.

[20] Egger M, Davey-Smith G, Altman D (2008). Systematic reviews in health care: meta-analysis in context.

[21] Erbay G, Sanli A, Turel H et al (2021) Short-term effects of COVID-19 on semen parameters: a multicenter study of 69 cases. Andrology. 10.1111/andr.13019 [published Online First: 15 Apr 2021] 10.1111/andr.13019 PMC8251422 33851521

[22] Ruan Y, Hu B, Liu Z (2021). No detection of SARS-CoV-2 from urine, expressed prostatic secretions, and semen in 74 recovered COVID-19 male patients: a perspective and urogenital evaluation. Andrology.

[23] Liu W, Han R, Wu H (2018). Viral threat to male fertility. Andrologia.

[24] Heller CG, Clermont Y (1963). Spermatogenesis in man: an estimate of its duration. Science.

[25] Xu J, Qi L, Chi X (2006). Orchitis: a complication of severe acute respiratory syndrome (SARS). Biol Reprod.

[26] Li N, Wang T, Han D (2012). Structural, cellular and molecular aspects of immune privilege in the testis. Front Immunol.

[27] Tajar A, Forti G, O'Neill TW (2010). Characteristics of secondary, primary, and compensated hypogonadism in aging men: evidence from the European Male Ageing Study. J Clin Endocrinol Metab.

[28] Grande G, Milardi D, Baroni S (2018). Identification of seminal markers of male accessory gland inflammation: from molecules to proteome. Am J Reprod Immunol.

[29] Lotti F, Corona G, Mancini M (2011). Ultrasonographic and clinical correlates of seminal plasma interleukin-8 levels in patients attending an andrology clinic for infertility. Int J Androl.

[30] Lotti F, Maggi M (2013). Interleukin 8 and the male genital tract. J Reprod Immunol.

[31] Vishvkarma R, Rajender S (2020). Could SARS-CoV-2 affect male fertility?. Andrologia.

