# Supplementary material for: Semen parameters in men recovered from COVID-19: a systematic review and meta-analysis
Source: Middle East Fertil Soc J. 2021 Dec 2;26(1):44. doi: 10.1186/s43043-021-00089-w (PMC8638229; doi:10.1186/s43043-021-00089-w)
Supplement: Supplementary file 1 — Additional file 1: Supplementary Table 1. Risk of bias assessment of included studies. [file 43043_2021_89_MOESM1_ESM.pdf]

Table 2. Risk of bias assessment of included studies

Author(Year)	Selection				Comparability	Outcome			Total	Quality
	1	2	3	4	1	1	2	3		
Cross-sectional studies										
Erbay (2021)	★		★	★	★	★	★		6	good
Ma (2021)	★		★		★	★		★	5	good
Raun (2021)		★		★	★	★	★	★	6	good
Temiz (2021)	★	★		★	★	★		★	6	good
Xu (2021)	★	★		★	★			★		good
Case-control studies										
Guo (2021)	★	★		★	★	★	★	★	7	good
Cohort studies										
Holtman (2021)	★	★		★	★	★	★	★	7	good
