# Supplementary material for: Semen parameters in men recovered from COVID-19: a systematic review and meta-analysis
Source: Middle East Fertil Soc J. 2021 Dec 2;26(1):44. doi: 10.1186/s43043-021-00089-w (PMC8638229; doi:10.1186/s43043-021-00089-w)
Supplement: Supplementary file 2 — Additional file 2: Supplementary Fig. 1. Funnel plot for pooling of semen volume. Supplementary Fig. 2. Funnel plot for pooling of sperm concentration. Supplementary Fig. 3. Funnel plot for total sperm number. Supplementary Fig. 4. Funnel plot for progressive sperm motility. Supplementary Fig. 5. Funnel plot for sperm motility. Supplementary Fig. 6. Funnel plot for Follicle stimulating hormone. Supplementary Fig. 7. Funnel plot for Luteinizing hormone. Supplementary Fig. 8. Funnel plot for Testosterone. [file 43043_2021_89_MOESM2_ESM.pdf]

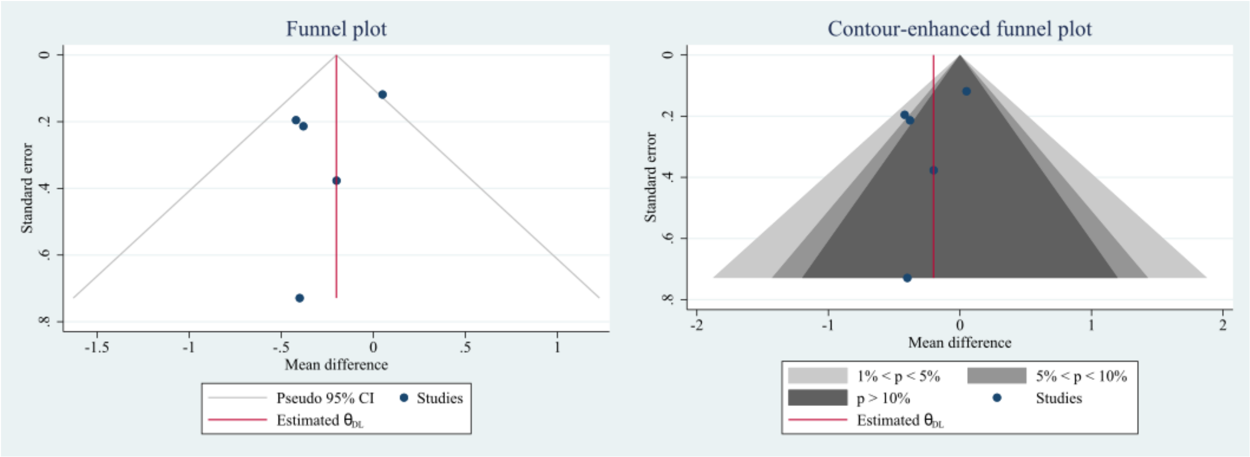

Figure 1. Funnel plot for pooling of semen volume

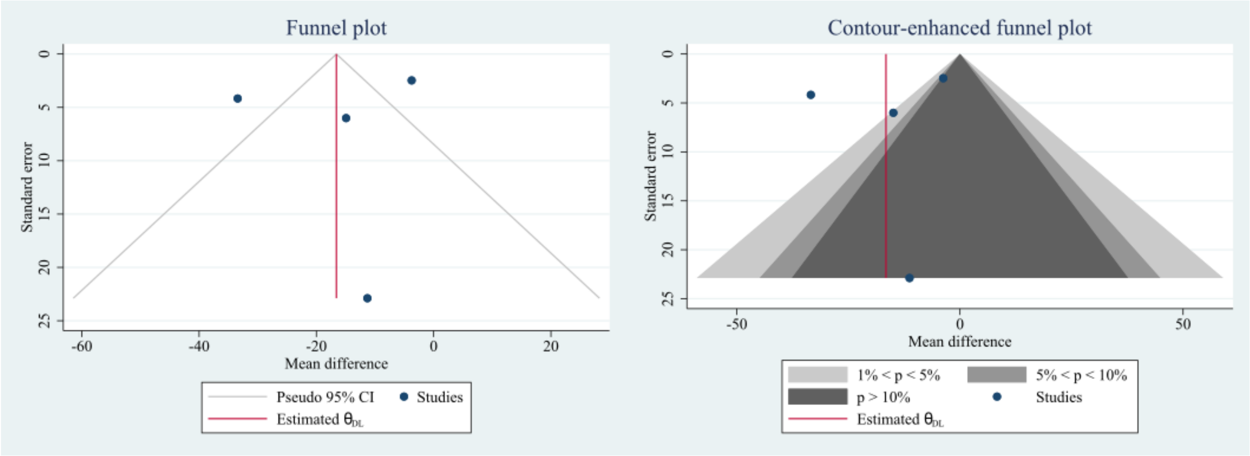

Figure 2. Funnel plot for pooling of sperm concentration

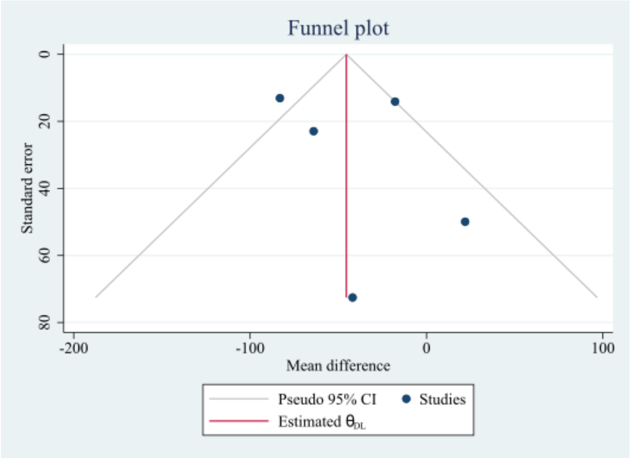

Figure 3. Funnel plot for total sperm number

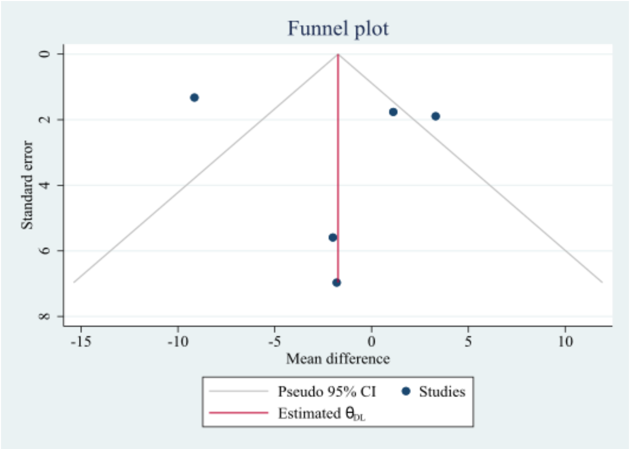

Figure 4. Funnel plot for progressive sperm motility

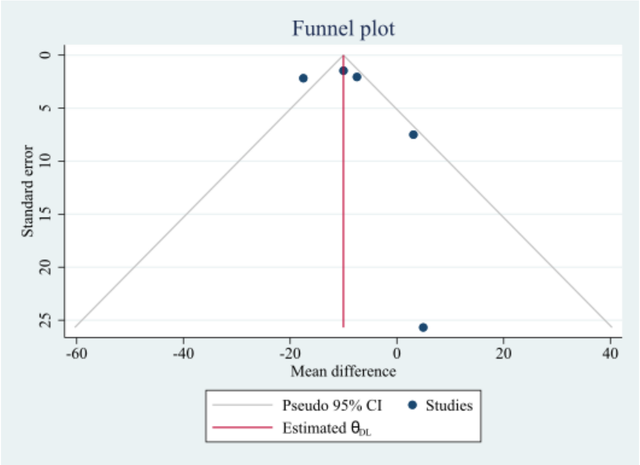

Figure 5. Funnel plot for sperm motility

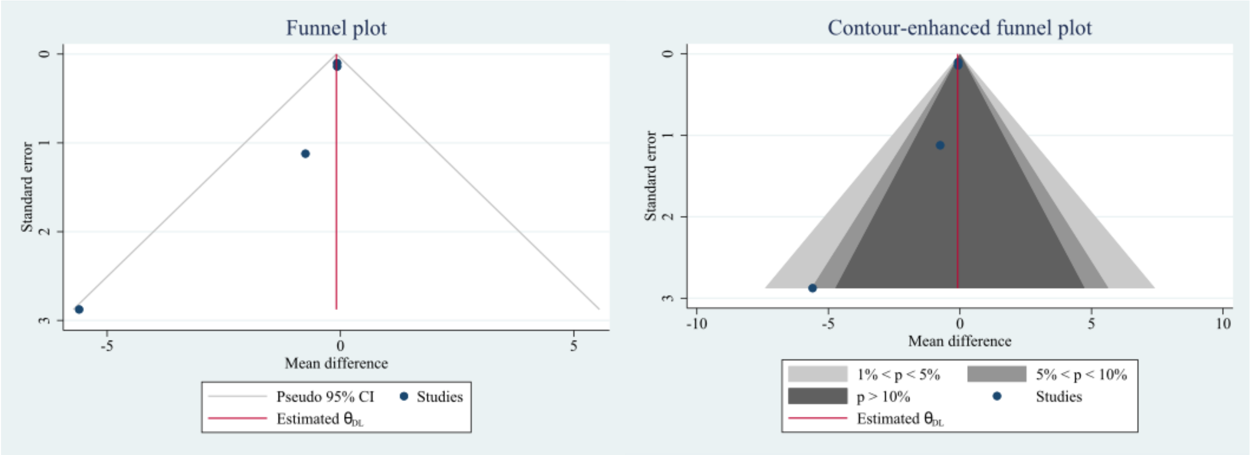

Figure 6. Funnel plot for Follicle stimulating hormone

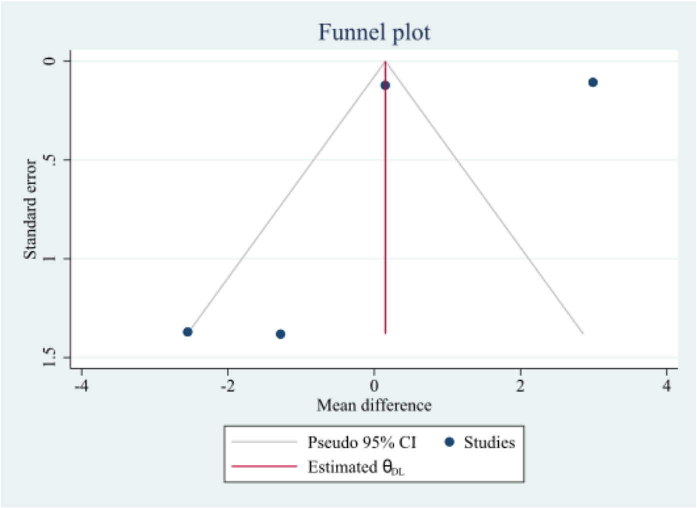

Figure 7. Funnel plot for Luteinizing hormone

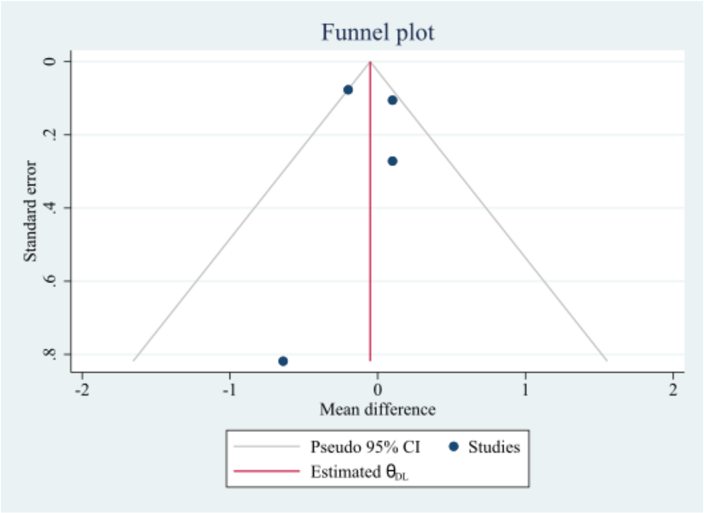

Figure 8. Funnel plot for Testosterone
